# Direct admission versus interhospital transfer for revascularisation in non‐ST‐segment elevation myocardial infarction

**DOI:** 10.1002/clc.24060

**Published:** 2023-06-21

**Authors:** Gijs J. van Steenbergen, Jesse P. A. Demandt, Daniela N. Schulz, Pim A. Tonino, Lukas Dekker, Iris Vermeer‐Gerritzen, Inge F. Wijnbergen, Eric J. M. Thijssen, Luc J. H. J. Theunissen, Eric P. C. M. Heijmen, Rutger J. P. Haest, Pieter‐Jan Vlaar, Dennis van Veghel

**Affiliations:** ^1^ Catharina Heart Centre Catharina Hospital Eindhoven The Netherlands; ^2^ Netherlands Heart Network (NHN) South‐East Brabant The Netherlands; ^3^ Centraal Ziekenfonds (CZ) Tilburg The Netherlands; ^4^ Maxima Medical Center Veldhoven The Netherlands; ^5^ Elkerliek Hospital Helmond The Netherlands; ^6^ St. Anna Hospital Geldrop The Netherlands

**Keywords:** cardiology, cardiothoracic surgery, ESC, NSTEMI, patient value, value‐based healthcare

## Abstract

**Background:**

The differences in outcomes and process parameters for NSTEMI patients who are directly admitted to an intervention centre and patients who are first admitted to a general centre are largely unknown.

**Hypothesis:**

There are differences in process indicators, but not for clinical outcomes, for NSTEMI who are directly admitted to an intervention centre and patients who are first admitted to a general centre.

**Methods:**

We aim to compare process indicators, costs and clinical outcomes of non‐ST‐segment elevation myocardial infarction (NSTEMI) patients stratified by center of first presentation and revascularisation strategy. Hospital claim data from patients admitted with a NSTEMI between 2017 and 2019 were used for this study. Included patients were stratified by center of admission (intervention vs. general center) and subdivided by revascularisation strategy (PCI, CABG, or no revascularisation [noRevasc]). The primary outcome was length of hospital stay. Secondary outcomes included: duration between admission and diagnostic angiography and revascularisation, number of intracoronary procedures, clinical outcomes at 30 days (MACE: all‐cause mortality, recurrent myocardial infarction and cardiac readmission) and total costs (accumulation of costs for hospital claims and interhospital ambulance rides).

**Results:**

A total of 9641 NSTEMI events (9167 unique patients) were analyzed of which 5399 patients (56%) were admitted at an intervention center and 4242 patients to a general center. Duration of hospitalization was significantly shorter at direct presentation at an intervention centre for all study groups (5 days [2–11] vs. 7 days [4–12], *p* < 0.001). For PCI, direct presentation at an intervention center yielded shorter time to diagnostic angiography (1 day [0–2] vs. 1 day [1–2], *p* < 0.01) and revascularisation (1 day [0–3] vs. 4 days [1–7], *p* < 0.001) and less intracoronary procedures per patient (2 [1–2] vs. 2 [2–2], *p* < 0.001). For CABG, time to revascularisation was shorter (8 days [5–12] vs. 10 days [7–14], *p* < 0.001). Total costs were significantly lower in case of direct presentation in an intervention center for all treatment groups €10.211 (8750–18.192) versus €13.741 (11.588–19.381), *p* < 0.001) while MACE was similar 11.8% versus 12.4%, *p* = 0.344).

**Conclusion:**

NSTEMI patients who were directly presented to an intervention center account for shorter duration of hospitalization, less time to revascularisation, less interhospital transfers, less intracoronary procedures and lower costs compared to patients who present at a general center.

## Graphical abstract

Patients directly admitted to intervention centers for non‐ST‐segment elevation myocardial infarction had shorter hospital stays and reduced costs compared to those admitted to general centers. However, logistical challenges and inconsistent clinical benefits suggest alternative approaches like improved prehospital triage and streamlined regional arrangements should be explored for optimizing resources and outcomes.

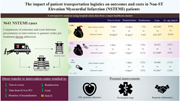

## 1 INTRODUCTION

Non‐ST‐segment elevation myocardial infarction (NSTEMI) is defined as angina symptoms with elevated cardiac biomarkers in the absence of persistent ST‐segment elevation.^1^ Based on numerous randomized trials and several meta‐analyses, a routine invasive strategy is recommended by the European society of Cardiology (ESC) guideline in most NSTEMI patients.^1^ Since 2015, the ESC has increasingly endorsed an early invasive strategy and in 2020, the most recent guidelines for myocardial revascularisation recommend same day transfer of patients with an established NSTEMI diagnosis from a general to an intervention center (class I, level of evidence A).^1^

However, in the Netherlands and other countries in Europe, only generic regional arrangements are made between centers and emergency medical services toward the location of patient presentation of NSTEMI patients.^2^, ^3^, ^4^, ^5^ The expert opinion of the paramedic in conjunction with telephonic consultation with the attending cardiologist (if deemed necessary), leads to a decision where to present a patient. In practise this means low and high risk NSTEMI patients are transferred to the nearest hospital (intervention or nonintervention center) and very high risk patients (i.e., haemodynamic instability or cardiogenic shock, recurrent or ongoing chest pain refractory to medical treatment, life‐threatening arrhythmias, mechanical complications of MI, acute heart failure, or recurrent dynamic ECG changes) are, in accordance with the ESC guideline and in analogy to the STEMI population, directly admitted to an intervention center.^6^, ^7^ Although we know that these recommendations are not always followed in clinical practise.^4^

Furthermore, in the Netherlands, intracoronary angiography (ICA) is performed at both general and intervention centers; however only the latter has on‐site revascularisation facilities (either PCI or CABG) due to national legislation. General centers, in contrast, are limited to diagnostic invasive facilities, without PCI or CABG capabilities. As a result, NSTEMI patients who are initially admitted at a general center and are candidates for revascularisation inevitably require interhospital transfer. The current situation in the Netherlands therefore lends itself to evaluate the incremental value of the ESC guideline recommendation of same day transfer of patients with an established NSTEMI diagnosis from a general to an intervention center. The primary objective of the current study is to conduct a comparative analysis of process indicators, clinical outcomes and costs of NSTEMI patients who are directly admitted at an intervention center and patients who are first admitted at a general hospital.

## 2 METHODS

### 2.1 Study design and study population

The design of the current study was a retrospective cohort study using aggregated hospital claim data from CZ (Centraal Ziekenfonds) which is one of the largest health insurance companies in the Netherlands with 3.7 million insured persons in 2019 and a market share of ~16% of the total population and 50% of the South of the Netherlands (in the Netherlands basic medical insurance is mandatory and all NSTEMI care is covered).^8^ The study was approved by the local medical ethical committee, which waived the need for informed consent (W20.310).

The study population consisted of adult patients who presented at an emergency department and were diagnosed by a physician with a NSTEMI (ICD‐10 I21.4) between 2017 and 2019 and were insured at CZ. Each unique NSTEMI event was included. Patients who switched to another health insurer within 30 days after the event were excluded (~6% of the insured switch annually). Patients who had a NSTEMI diagnosis registration during an existing admission for another reason were excluded because the aim of the current study focusses on logistics in NSTEMI hospitalizations.

Alongside an analysis of the total study population, a division was made for each NSTEMI event into the following cohorts:

(1) NSTEMI events for which patients underwent PCI during initial admission;
(2) NSTEMI events for which patients underwent CABG during initial admission;
(3) NSTEMI events for which patients did not undergo revascularisation during initial admission (noRevasc).

For the total population and each cohort, a distinction was made between presentation at an intervention center and presentation at a general center. A hospital was considered an intervention center if facilities for performing PCI and/or CABG were available on site. A general center was defined as a center that only has diagnostic invasive facilities, and does not have PCI or CABG facilities. An admission was defined as the uninterrupted, cumulative clinical episodes in intervention and general centers.

### 2.2 Baseline characteristics

Given the utilization of a database based on insurance claim data, a comprehensive characterization of the study population is not anticipated. This stems from the fact that not all findings corresponding to an NSTEMI are reported to the insurance provider. Nevertheless, to facilitate a scoping understanding of the patient population under study, several characteristics can be inferred from the available claim and diagnosis records. As such, the following baseline characteristics were assessed for each NSTEMI event in the study period: age, gender, diabetes mellitus, obstructive pulmonary disease (COPD/asthma), renal insufficiency, previous PCI and previous CABG. Data was based on historical claim data from CZ and was available from 2014 onward. Diabetes mellitus (DM) was defined as a diagnosis registration on a hospital reimbursement claim for DM (or its complications) or use of glucose‐lowering medication. Obstructive pulmonary disease was defined as a diagnosis registration on a hospital reimbursement claim for COPD/asthma or use of any inhalation medication. Renal insufficiency, previous PCI and previous CABG were defined as a historical registered diagnosis for the disease or treatment.

### 2.3 Study outcomes

The primary outcome for this study was duration of hospitalization. This outcome reflects the NSTEMI care pathway most accurately and may act as a surrogate for time to invasive diagnostic work‐up and revascularisation. The latter are of importance to improve outcomes of NSTEMI patients.^1^

Secondary outcomes were process indicators (time from admission to ICA and revascularisation and total number of coronary diagnostic sessions performed during admission), costs and MACE at 30 days.

A coronary diagnostic session was defined as all types of diagnostic procedures (ICA, Fractional Flow Reserve; FFR, Optical Coherence Tomography; OCT, and/or Intravascular Ultrasound; IVUS) performed during the same session.

Cost were calculated based on costs for hospital claims and ambulance rides during admission. Costs of hospital claims were based on the Diagnosis Treatment Combinations (in Dutch: Diagnose Behandel Combinatie; DBC). In the Netherlands, hospitals are paid for provided care through DBCs which consists of care activities standard for a disease or treatment within a predefined period. The price of the DBC therefore represent the average costs provided for a disease or treatment independent of the risk profile of the individual patient. Physicians initiate a DBC by registering a diagnosis. Because each hospital in the Netherlands makes their own price agreements with insurance companies and receive imbursement for provided care, patients who require interhospital transfer can have multiple DBCs for the same diagnosis if care is provided at different hospitals. The average cost price per ambulance ride and hospital claim in the Netherlands was used for this study.

MACE was defined as a composite of all‐cause mortality within 30‐day after the NSTEMI event, myocardial infarction (STEMI or NSTEMI) within 30 days after discharge and cardiac readmission rate (defined as all readmission for cardiovascular causes excluding readmissions for myocardial infarction) at 30 days using claim data from CZ. These outcomes were also reported individually. Mortality was subdivided in mortality between 0 and 5 days and 6–30 days because differences in mortality between these periods may be the result of triage that we cannot identify from the baseline data.

### 2.4 Statistical analyses

General descriptive statistics of included NSTEMI events were used to describe baseline characteristics and primary and secondary outcomes as stratified by the three treatment modalities and subdivided per hospital of presentation. Data was expressed as mean ± standard deviation (SD) for continuous normal distributed data, median with interquartile range (IQR) for continuous non‐normal distributed data and as absolute and relative frequencies for categorical data. Student's *t* test, Mann–Whitney *U* test or Fisher exact‐test/*χ* ^2^ test respectively were used to make a comparison between cohorts where appropriate. A *p* < 0.05 was considered significant and all analyses were performed using SPSS 25 (SPSS Inc).

## 3 RESULTS

**Figure 1:**
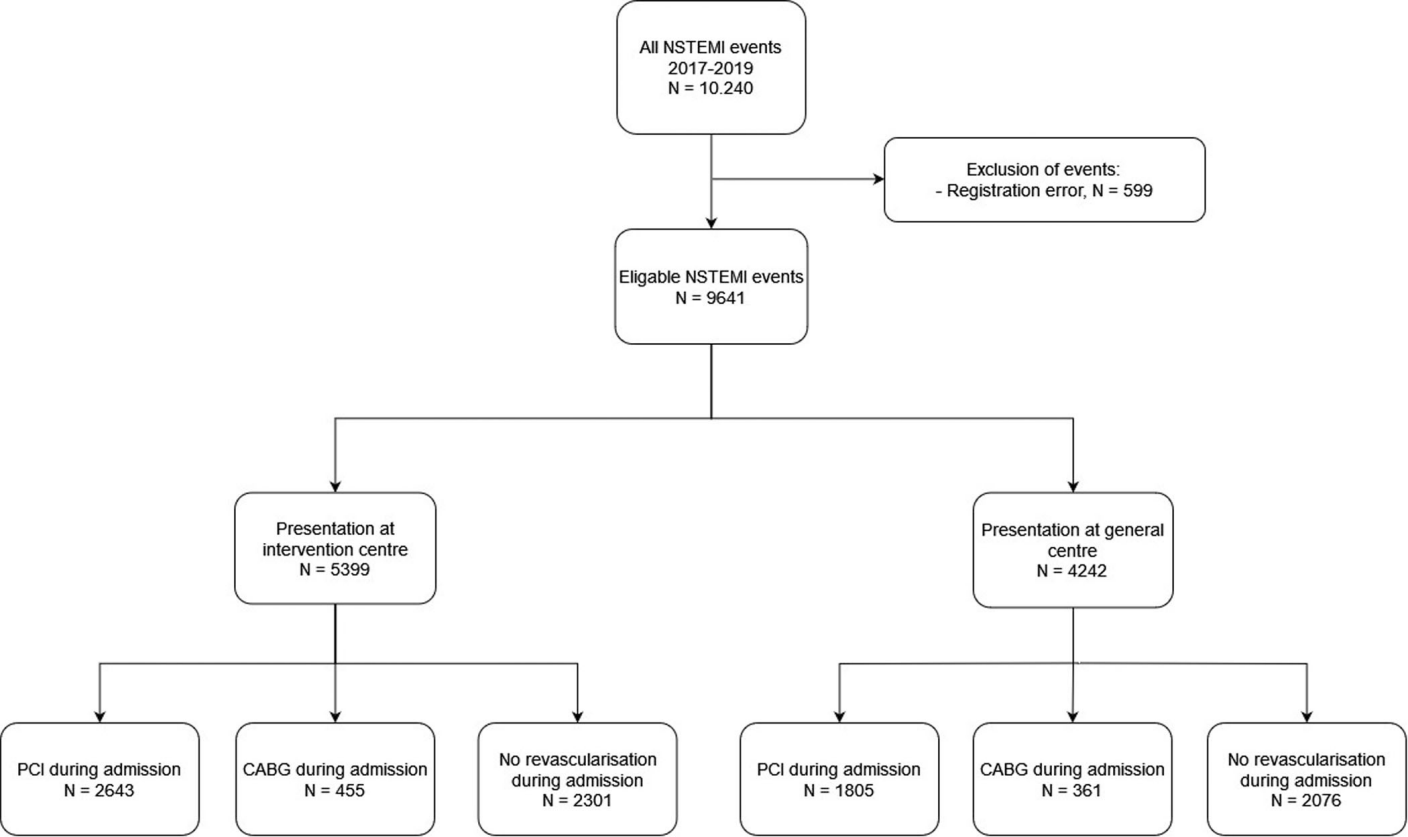
Flowchart of included patients and allocation to study groups. CABG, coronary artery bypass surgery; NSTEMI, non–ST‐segment elevation myocardial infarction; PCI, percutaneous coronary intervention.

After applying the inclusion and exclusion criteria, 9641 NSTEMI events in 9167 unique patients were eligible for inclusion. A total of 5399 patient first presented at an intervention center (56% of total) and 4242 patients at a general center. PCI was performed during admission to treat 4448 event (2643 vs. 1805 events respectively), CABG was performed for 816 events (455 vs. 361 events) and 4377 event (2301 vs. 2076 events) were treated without revascularisation during admission (noRevasc; Figure 1). In the total population, no significant difference in baseline characteristics was found between study groups (Table 1).

**Table 1:** Baseline characteristics associated with unique NSTEMI events stratified by treatment during admission and center of initial presentation.

	Total			PCI			CABG			NoRevasc		
	*N* = 9641			*N* = 4448			*N* = 816			*N* = 4377		
	Intervention center	General hospital	*p* Value	Intervention center	General hospital	*p* Value	Intervention center	General hospital	*p* Value	Intervention center	General hospital	*p* Value
	*N* = 5399	*N* = 4242		*N* = 2643	*N* = 1805		*N* = 455	*N* = 361		*N* = 2301	*N* = 2076	
Gender‐male	3507 (65.0)	2709 (63.9)	0.274	1865 (70.6)	1271 (70.4)	0.920	373 (82.0)	298 (82.5)	0.854	1269 (55.1)	1140 (54.9)	0.879
Age	70 (60–78)	70 (60–79)	0.210	68 (58–77)	68 (59–77)	0.506	67 (60–73)	68 (59–75)	0.358	73 (62–82)	73 (62–82)	0.407
Diabetes Mellitus	1590 (29.4)	1209 (28.5)	0.309	709 (26.8)	464 (25.7)	0.426	130 (28.6)	102 (28.3)	0.938	751 (32.5)	643 (31.0)	0.242
Chronic pulmonary disease	1290 (23.9)	1011 (23.8)	0.962	561 (21.2)	406 (22.5)	0.166	74 (16.3)	56 (15.5)	0.424	655 (28.5)	549 (26.4)	0.072
Renal insufficiency	304 (5.6)	226 (5.3)	0.529	117 (4.4)	60 (3.3)	0.037	16 (3.5)	12 (3.3)	0.520	171 (7.4)	154 (7.4)	0.517
Previous PCI	1141 (21.1)	919 (21.7)	0.532	538 (20.4)	355 (19.7)	0.300	53 (11.6)	52 (14.4)	0.144	550 (23.9)	512 (24.7)	0.291
Previous CABG	90 (1.7)	60 (1.4)	0.362	44 (1.7)	23 (1.3)	0.178	1 (0.2)	2 (0.6)	0.414	45 (2.0)	35 (1.7)	0.291

*Note*: Data are presented as median (IQR) or *n* (%).

Abbreviations: CABG, coronary artery bypass surgery; NoRevasc, no invasive treatment; PCI, percutaneous coronary intervention.

### 3.1 Primary outcome

For the total study population, length of stay was shorter for patients directly admitted to an intervention center in comparison to initial admission to a general center (5 days, IQR 2–11 vs. 7 days, IQR 4–12, *p* < 0.001). A similar finding was found for patients who underwent PCI (3 days, IQR 2–6 vs. 5 days, IQR 3–8, *p* < .001), CABG (16 days, IQR 12–21 vs. 18 days, IQR 14–23, *p* < 0.001) and in patients who underwent no revascularisation during admission (3 days, IQR 2–6 vs. 4 days, IQR 2–6, *p* = 0.002, Table 2).

**Table 2:** Primary and secondary outcomes related to unique NSTEMI events stratified by center of presentation and treatment strategy during initial admission.

	Total population			PCI			CABG			NoRevasc		
	*N* = 9641			*N* = 4448			*N* = 816			*N* = 4377		
	Intervention center	General hospital	*p* Value	Intervention center	General hospital	*p* Value	Intervention center	General hospital	*p* Value	Intervention center	General hospital	*p* Value
	*N* = 5399	*N* = 4242		*N* = 2643	*N* = 1805		*N* = 455	*N* = 361		*N* = 2301	*N* = 2076	
Primary outcome												
Length of stay, median (IQR) days	5 (2–11)	7 (4–12)	<0.001	3 (2–6)	5 (3–8)	<0.001	16 (12–21)	18 (14–23)	<0.001	3 (2–6)	4 (2–6)	0.002
Secondary outcomes												
Time from admission to ICA, median (IQR) days	1 (0–2)	1 (1–2)	<0.001	1 (0–2)	1 (1–2)	<0.001	1 (0–3)	1 (1–3)	0.348	1 (1–3)^a^	1 (1–3)^a^	0.236
ICA < 24 h, *n* (%)	1908 (58.3)	1636 (56.1)	0.089	977 (63.1)	840 (59.2)	0.032	246 (57.3)	193 (56.9)	0.942	685 (52.9)	603 (52.1)	0.716
ICA < 72 h, *n* (%)	2768 (84.6)	2540 (87.2)	0.004	1345 (86.9)	1255 (88.5)	0.199	361 (84.1)	299 (88.2)	0.118	1062 (81.9)	986 (85.2)	0.029
Time from admission to revascularisation, median (IQR) days	2 (0–6)	6 (3–9)	<0.001	1 (0–3)	4 (1–7)	<0.001	8 (5–12)	10 (7–14)	<0.001	‐	‐	‐
Performed <24 h, *n* (%)	1649 (53.2)	596 (27.5)	<0.001	1595 (60.3)	579 (32.1)	<0.001	54 (11.9)	17 (4.7)	<0.001	‐	‐	‐
Performed <72 h, *n* (%)	2225 (71.8)	901 (41.6)	<0.001	2140 (81.0)	870 (48.2)	<0.001	85 (18.7)	31 (8.6)	<0.001	‐	‐	‐
Unique procedures performed^b^, median (IQR)	2 (2–2)	2 (2–2)	0.090	2 (1–2)	2 (2–2)	<0.001	2 (2–2)	2 (2–2)	0.672	1 (0–1)	1 (0–1)	0.197
FFR, IVUS or OCT, *n* (%)	211 (3.9)	94 (2.2)	<0.001	187 (7.1)	81 (4.5)	<0.001	1 (0.1)	‐	1.00	23 (0.6)	13 (0.8)	0.184
Total costs, median (IQR)	€10.211 (8750–18.192)	€13.741 (11.588–19.381)	<0.001	€9.472 (8.325–11.001)	€12.908 (10.963–14.983)	<0.001	€22.995 (21.358 – 25.837)	€25.707 (23.372–29.052)	<0.001	€4.886 (3.725–7.499)	€5.204 (3.742–7.986)	0.001
Ambulance costs	€749 (364–965)	€1205 (831–1675)	<0.001	€742 (0–816)	€1263 (871–1735)	<0.001	€890 (690–1553)	€1230 (750–1706)	<0.001	€737 (0–794)	€740 (0–865)	0.033
Number of rides	1 (1–2)	2 (2–3)	<0.001	1 (0–1)	2 (2–3)	<0.001	2 (1–3)	2 (2–3)	<0.001	1 (0–1)	1 (0–2)	<0.001
Costs for hospital claims	€9585 (8325–17.275)	€12.475 (10.475–17.660)	<0.001	€8.720 (8.245–10.201)	€11.700 (9.687–13.505)	<0.001	€22.270 (20.900–24.390)	€24.480 (22.255–27.822)	<0.001	€4.150 (2.980 – 6.805)	€4.575 (3.160–7.196)	<0.001
Number of claims	3 (2–4)	4 (3–5)	<0.001	2 (2–3)	4 (3–5)	<0.001	4 (3–5)	5 (4–6)	<0.001	2 (1–2)	2 (1–3)	<0.001
MACE, *n* (%)	635 (11.8)	526 (12.4)	0.344	244 (9.2)	163 (9.0)	0.832	42 (9.2)	39 (10.8)	0.481	349 (15.2)	324 (15.6)	0.706
All‐cause 30‐day mortality	202 (3.7)	161 (3.8)	0.914	52 (2.0)	17 (0.9)	0.006	9 (2.0)	5 (1.4)	0.596	141 (6.1)	139 (6.7)	0.458
0–5 days mortality	90 (1.7)	45 (1.1)	0.014	23 (0.9)	6 (0.3)	0.024	3 (0.7)	1 (0.3)	0.633	64 (2.9)	38 (2.1)	0.116
6–30 day mortality	112 (2.1)	116 (2.7)	0.635	29 (1.1)	11 (0.6)	0.087	6 (1.3)	4 (1.1)	0.596	77 (3.4)	101 (4.6)	0.058
Myocardial infarction at 30 days	112 (2.1)	107 (2.5)	0.112	37 (1.4)	34 (1.9)	0.207	2 (0.4)	2 (0.3)	0.587	69 (2.9)	76 (3.7)	0.201
Cardiac readmissions at 30 days^c^	338 (6.3)	265 (6.2)	1.00	161 (6.1)	114 (6.3)	0.390	33 (7.3)	32 (8.9)	0.372	144 (6.3)	119 (5.7)	0.876

Abbreviations: CABG, coronary artery bypass surgery; ICA, intracoronary angiography.

^a^ ICA was performed in 56.3% and 55.7% of patients for an intervention or general center respectively.

^b^ Consists of CABG, ICA, PCI, FFR, IVUS, OCT.

^c^ Excludes readmission for myocardial infarction.

### 3.2 Secondary outcomes

In the total study population, median time from admission to ICA (1 day, IQR 0–2 vs. 1 day, IQR 1–2, *p* < 0.001), median time from admission to revascularisation (2 days, IQR 0–6 vs. 6 days, IQR 3–9, *p* < 0.001) and total costs (€10.211, IQR 8750–18.192 vs. €13.741, IQR 11.588–19.381, *p* < 0.001) were significantly different in favor of patients directly admitted to an intervention center (Table 2). MACE and mortality were not significantly different (11.8% vs. 12.4%, *p* = 0.344 and 3.7% and 3.8%, *p* = 0.914, respectively).

In the PCI cohort, time from admission to ICA (1 day, IQR 0–2 vs. 1 day, IQR 1–2, *p* < 0.001) and time from admission to revascularisation (1 day, IQR 0–3 vs. 4 days, IQR 1–7, *p* < 0.001) were shorter in an intervention center compared to a general hospital. A statistical significant difference was found in favor of intervention centers in terms of ICAs performed within 24 h (63.1% vs. 59.2%, *p* = 0.032), but not within 72 h (86.9% vs. 88.5%, *p* = 0.199). FFR, IVUS or OCT was performed scarcely but more often if patients were admitted directly at an intervention center. No difference was found in MACE (9.2% vs. 9.0%, *p* = 0.832). All‐cause 30‐day mortality was higher in an intervention compared to a general center (2.0% vs. 0.9%, *p* = 0.006). All‐cause mortality in the first 5 days was 0.9% versus 0.3% (*p* = 0.024) and for 6–30 days 1.1% versus 0.6% (*p* = 0.087). Costs were lower for patients directly transferred to an intervention center (€9.472, IQR 8325–11.001 vs. €12.908, IQR 10.963–14.983, *p* < 0.001).

In the CABG cohort, no difference was found between the hospitals in time from admission to ICA (1 day, IQR 0–3 vs. 1 day, IQR 1–3, *p* = 0.348). Direct admission to an intervention resulted in shorter time from admission to revascularisation in comparison to direct admission at a general hospital (8 days, IQR 5–12 vs. 10 days, IQR 7–14, *p* < 0.001). MACE was similar between centers (9.2% vs. 10.8%, *p* = 0.481). Costs were lower for patients directly admitted at an intervention center (€22.995, IQR 21.358–25.837 vs. €25.707, IQR 23.372–29.052, *p* < 0.001)

In patients who did not undergo revascularisation during admission, ICA was performed in 56.3% and 55.7% of patients at an intervention or general center, respectively. No difference was found in time from admission to ICA (1 day, IQR 1–3 vs. 1 day, IQR 1–3, *p* = 0.236). In a general hospital, more ICAs were performed within 72 h compared to an interventional center (85.2% vs. 81.9%, *p* = 0.029). No difference in MACE was found (15.2% vs. 15.6%, *p* = 0.706). Costs were lower for patients directly admitted to an intervention center (€4886, IQR 3725–7499 vs. €5207, IQR 3742–7986, *p* < 0.001, Table 2).

## 4 DISCUSSION

The principal finding of the current study was that patients who were directly admitted to an intervention center had shorter duration of hospitalization compared to patients first admitted to a general center regardless if these patients undergo revascularisation during admission or not. This prolonged duration of hospitalization appeared driven by longer time between admission and intracoronary angiography (ICA) in PCI patients while the duration between admission and the PCI and CABG also contributed to these differences. Additionally, secondary outcomes revealed that patients requiring revascularization and initially admitted to a general center had significantly more interhospital ambulance rides and hospital claims, leading to higher overall costs. The current logistics in NSTEMI care in the Netherlands hereby show to introduce significant delay to invasive treatment, suboptimal use of healthcare resources and significant expenses which are burdensome to patients and society.

In general, there is limited previous data published on outcomes of NSTEMI patients admitted at a general versus an intervention center. A subgroup analysis of the ELISA‐3 trial on timing of intervention in high‐risk NSTEMI patients in PCI versus non‐PCI centers found that patients initially hospitalized in non‐PCI centers show the largest benefit from early ICA and revascularisation (relative risk 0.23 vs. 0.85 for incidence of the combined primary endpoint of death, reinfarction and recurrent ischemia after 30 days follow‐up) and a shorter waiting time to revascularisation (4–7 days).^9^ Furthermore, as part of an evaluation in 2020 by the national audit for PCI in the United Kingdom, delay to PCI in NSTEMI patients prolonged with 24 h if patients required interhospital transfer compared to direct presentation at a PCI center.^10^ A sub analysis of EARLY‐ACS Trial by Toleva et al.^11^ which evaluated outcomes in 9204 patients presenting to tertiary sites, primary sites with transfer to tertiary sites (“transferred”) and those who remained at primary sites (“non‐transfer”) found that timely angiography and revascularization were often not achieved in transferred patients. Toleva et al.^11^ also concluded significant delays occurred in time from symptom onset to angiography (49 h), PCI (53 h), and CABG (178 h) for transferred patients (*p* < 0.001) which also corroborates with our findings.

The principal findings of our study at first may seem to support the most recent ESC recommendation that all patients with an established NSTEMI diagnosis should have same‐day transfer to an intervention center.^1^ Nevertheless, transferring all patients with an established NSTEMI diagnoses directly to an intervention center yields logistics challenges, despite the apparent reduction in costs, and several considerations should be taken into account. First, the effect of an early invasive strategy (<24 h) on clinical outcomes is not yet well established for all NSTEMI patients.^7^, ^12^ A recent meta‐analysis showed early invasive treatment did not reduce mortality and morbidity but might reduce risk of recurrent ischemia only in a subgroup of high risk patients.^9^, ^13^, ^14^, ^15^ Our study showed similar MACE and 30‐day mortality in the total population. This is important as this finding is based on real‐world data analyses and ads valuable information to the current (limited) data available. However, the subgroup of patients who underwent PCI had a significantly higher 30‐day mortality in patients directly admitted at an intervention center. The existing triage of very‐high risk patients to an intervention center is the only plausible reason for these differences in mortality.^2^, ^3^, ^4^, ^5^ Furthermore, prehospital triage based on clinical profile is also highlighted by the significant different mortality for PCI only in the first 5 days (and not in the remaining days). Second, in the current study, not all patients (~50%) who are diagnosed with a NSTEMI at presentation undergo ICA which was also demonstrated in a study by Hoedemakers et al.^4^ As such, centralization to intervention centers might demand capacity adjustments in intervention centers while not all patients undergo ICA or revascularisation. Furthermore, this could potentially yield a loss of expertise and catheterization skills in general centers.

Thus, one could speculate on alternative adjustments to NSTEMI logistics that might require less radical changes. First, more accurate prehospital triage with use of risk scores that stratify chest pain patients in “risk” categories for having a NSTEMI (e.g., preHEART and the modified HEART) might improve patient allocation and most efficient use of resources.^16^, ^17^ Some experience has been described with direct access pathways in London. The Royal Free Hospital managed to reduce median time to ICA through this pathway to 2.8 h (IQR 1.5–9) compared to 60 h (IQR 33–116) with use of a pathway similar to the pathway evaluated in our study.^2^ Additionally, a two recently conducted randomized trials by Camaro et al.^18^ and Dawson et al.^19^ demonstrated that prehospital rule‐out of ACS with a point‐of‐care troponin measurement in chest pain patient's notably reduced healthcare expenditure. However, there is not yet consensus on the most ideal form of prehospital triage as several methods are still in the early stages of development and trials are currently being conducted.^20^ One such example is the highly anticipated TRIAGE‐ACS study which will be the first prospective study to investigate the impact of prehospital triage using the PreHEART score on time to final invasive diagnostics and treatment in patients with NSTE‐ACS in need of revascularization by transferring high risk patients directly to a PCI center and patients at a low risk of having an NSTE‐ACS to a non‐PCI center.^20^

Furthermore, the general efficiency of workflow and regional arrangements also yield improvement potential. Attributes to the primary findings are most probably ad hoc scheduling issues at the intervention center combined with heart team deliberation in selected patients (e.g., postponement of treatment decision due to missing information). Improvement potential could potentially lie in streamlining work processes with all regional actors and tailored scheduling. The application of information technologies for fast sharing of ICA data (which is available for most centers in the Netherlands) is a great step in this direction. Applying bundled payment models for the entire care chain associated with a NSTEMI event could also stimulate optimization of regional logistics and make providers financially accountable for the quality and costs of an entire NSTEMI episode. Also, these models could prevent duplicate claims by multiple providers.^21^, ^22^

### 4.1 Limitations

The current study is subject to general shortcomings associated with retrospective studies and relies on the correct diagnosis registration. Nevertheless, there is vast experience with using declaration or diagnosis registration data to evaluate outcomes for various conditions or treatments and this data is considered highly accurate.^23^ However, important confounding factors that are relevant for risk classification and adjustment were not available (i.e., hemodynamic parameters, findings on the ECG, lab results), thus impeding more in‐depth analysis such as regression analysis for clinical outcomes and risk stratification. It is worth noting that, aside from age, all other variables crucial to the GRACE risk score—a primary risk stratification tool for estimating mortality risk post‐NSTEMI—were unattainable.^24^ Future research with these variables at hand is warranted.

## 5 CONCLUSION

In conclusion, our study demonstrates that NSTEMI patients who are directly admitted to an intervention center experience shorter hospitalization durations, fewer interhospital transfers, and reduced costs compared to those first admitted to a general center. Despite these advantages, transferring all NSTEMI patients directly to intervention centers may pose logistical challenges and may not yield consistent clinical benefits across all patient subgroups. Exploring alternative approaches, such as enhancing prehospital triage and streamlining regional arrangements, is crucial for optimizing healthcare resources and improving patient outcomes. Further research is needed to validate these findings and investigate innovative strategies in various healthcare settings.

## AUTHOR CONTRIBUTIONS

*Planning*: Gijs J. van Steenbergen, Daniela N. Schulz, Dennis van Veghel, Lukas Dekker, Pim A. Tonino, and Iris Vermeer‐Gerritzen. *Conduct*: Gijs J. van Steenbergen, Dennis van Veghel, Iris Vermeer‐Gerritzen, Jesse P. A. Demandt, Lukas Dekker, and Pieter‐Jan Vlaar. *Reporting*: Gijs J. van Steenbergen, Jesse P. A. Demandt, Daniela N. Schulz, Pim A. Tonino, Lukas Dekker, Iris Vermeer‐Gerritzen, Inge F, Wijnbergen, Eric J. M. Thijssen, Luc J. H. J. Theunissen, Eric P. C. M. Heijmen, Rutger J. P. Haest, Pieter‐Jan Vlaar, and Dennis van Veghel. *Guarantors*: Dennis van Veghel, Lukas Dekker, and Pieter‐Jan Vlaar.

## CONFLICT OF INTEREST STATEMENT

The authors declare no conflict of interest.

## Data Availability

Data will become available to interested investigators upon submitting a reasonable research from corresponding author request by email.
